# Supplementary material for: Consensus on Prioritisation of Actions for Reducing the Environmental Impact of a Large Tertiary Hospital: Application of the Nominal Group Technique
Source: Int J Environ Res Public Health. 2023 Feb 23;20(5):3978. doi: 10.3390/ijerph20053978 (PMC10001469; doi:10.3390/ijerph20053978)
Supplement: Supplementary file 1 [file ijerph-20-03978-s001.zip › ijerph-2207863-supplementary.pdf]

Supplementary Materials

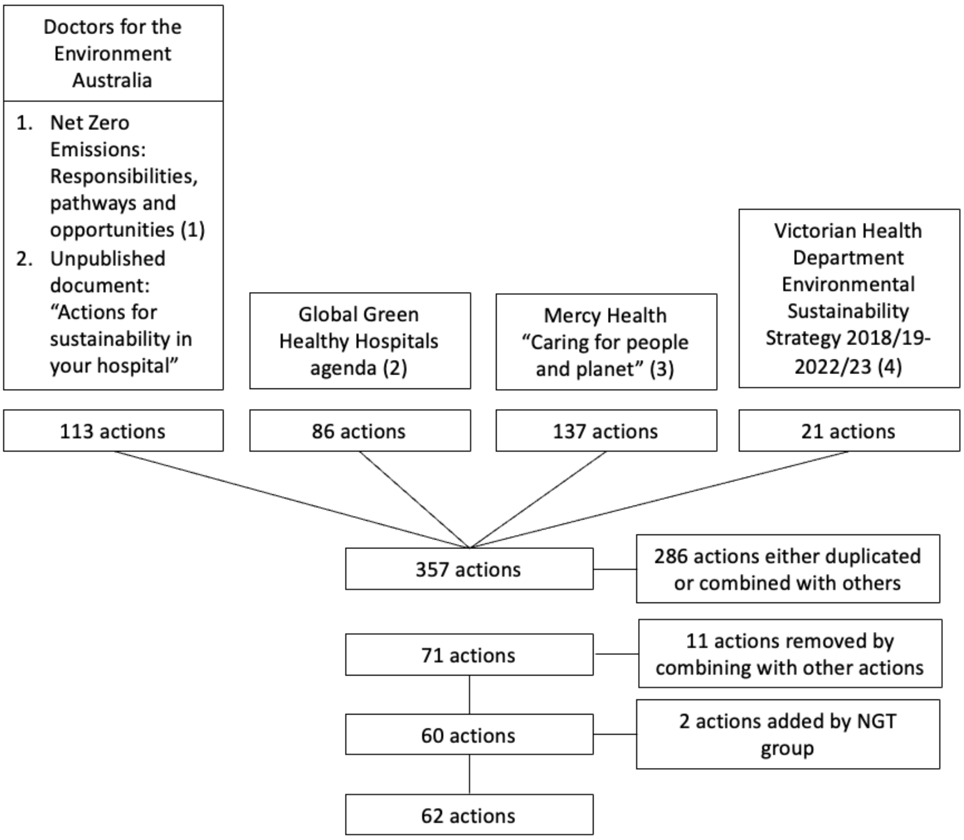

Figure S1. Process diagram for the development of the priority action list.

1. *Net Zero Carbon Emissions: Responsibilities, Pathways and Opportunities for Australia's Healthcare Sector*; Doctors for the Environment Australia: Carlton, Australia, 2020
2. *Global Green and Healthy Hospitals: A Comprehensive Environmental Health Agenda for Hospitals and Health Systems Around the World*; Healthcare Without Harm: Washington, DC, USA, 2011.
3. Mercy Health. *Caring for People and Planet: Mercy Health's Strategic Response to Laudato Si' 2020–25*; Mercy Health: Victoria, Australia, 2022.
4. Department of Health & Human Services. *Environmental Sustainability Strategy 2018–19 to 2022–23*; State Government of Victoria: Melbourne, Australia, 2022. Available online: https://www.health.vic.gov.au/publications/environmental-sustainability-strategy-2018-19-to-2022-23 (accessed on 17 October 2018).

Table S1. All ranked potential actions staff at a tertiary Australian hospital could take to improve the environmental sustainability of the health service, means of each score and the combined ranking of each summed score for amenability to change and for climate impact.

Final Rank	Proposed Priority Actions	Amenability to Change Summed Ratings (A) [n=11, possible score range 11–33]<br>Mean score (1–3) [High=3 Medium=2 Low=1]	Climate Impact Summed Ratings (B) [n=11, possible score range 11–33]<br>Mean score (1–3) [High=3 Medium=2 Low=1]	Ranking Total Score (A × B)
**1**	Engage hospital communications team to increase messaging around sustainability, promote local initiatives and broadcast any wins/successes in order to build momentum	32<br>3.0	28<br>2.3	896
**2**	Expand telehealth supports between Austin teams and referring health services to decrease the need for transfers and promote facilitation of care at regional/rural facilities	29<br>2.5	29<br>2.7	841
**3**<br>**4**	Include sustainability clauses in procurement contracts, encourage supplier 'take-back' programs for waste, a commitment from suppliers to achieve carbon neutrality	28<br>2.7	29<br>2.5	812
	Reduce patient travel with improved technology to support telemedicine and appointment bundling	27<br>2.7	30<br>2.5	810
**5**	Establish local area green groups led by local area green champions to coordinate initiatives across areas, share resources and educate	27<br>2.6	30<br>2.4	810
**5**<br>**6**	Commit to exclude fossil fuels (including natural gas) within planned capital upgrades by making capital upgrades electric only	29<br>2.6	27<br>2.6	783
	Establish education of the workforce on the health impacts of climate change and on low-carbon care pathways	26<br>2.8	30<br>2.1	780
**7**	Printing: double sided printing as standard on all printer settings, mandate paper sources to be ethically and sustainably sourced, work towards models of care that are paperless	26<br>2.5	30<br>2.4	780
**7**	Lights off at night program: Reduce energy usage after hours including clinical areas with low night-time activity and offices	31<br>2.8	21<br>2.5	775
**8**<br>**9**	Implement and prioritise reusable equipment in place of single-use equipment in operating theatres, ED, ICU and wards where possible	27<br>2.5	28<br>2.4	756
	Reduce environmental hazards by auditing use of Hydrofluorocarbons (HFCs), Sulfa hexafluorides (SF6s) and explore the possibility to reduce, prevent wastage or replacement	26<br>2.5	29<br>2.4	754
**10**<br>**10**	Initiate energy power-downs of high-energy usage areas such as heating ventilation and cooling (HVAC) in theatres during times of reduced activity such as nights and weekends	29<br>2.3	26<br>2.7	754
	Detect and remove leaks from nitrous oxide infrastructure (i.e. in theatres and emergency department)	25<br>2.7	30<br>2.3	750
**11**<br>**11**	Advocate for fossil-fuel free (all electric), zero emission and high-efficiency approaches to all new capital works	30<br>2.5	25<br>2.7	750
	Consider strategic sustainability research projects that will lead to financial and environmental savings for the hospital	27<br>2.5	27<br>2.5	729
**12**<br>**12**	Coordinate a triple-bottom-line decision-making policy for introducing new single-use equipment, through liaison with infection control, procurement and stakeholders	27<br>2.3	27<br>2.4	729
	Reduce energy use by installing motion-controlled lighting, occupancy sensors, LED lighting	26<br>2.5	28<br>2.4	728

**13**	Reduce single use disposable plastics in embedded retail	26<br>2.5	28<br>2.6	728
**13**<br>**14**	Adopt a strategy to reduce/replace fossil fuel including natural gas use within existing buildings, and where they cannot be immediately replaced optimise their efficiency	29<br>2.5	25<br>2.6	725
	Mandate that all bidding contractors have net zero approach to capital works	30<br>2.2	24<br>2.5	720
**15**	Adopt formal education strategies regarding environmental impact of clinical and occupational decisions. Include this in mandatory training.	24<br>2.6	30<br>2.6	720
**15**<br>**16**	Initiate sustainable pharmaceutical procurement for example, by preferencing companies with a net zero emissions target	27<br>2.2	25<br>2.1	675
	Dedicate education to recycling, establish mechanisms to feedback recycling and waste management activities (i.e. contamination rates) and update recycling educational resources	24<br>2.5	28<br>2.3	672
**17**	Negotiate smaller/less packaging with purchase of consumables	28<br>2.5	24<br>2.5	672
**17**<br>**18**	Embed sustainability into research plans and funding such as requiring environmental assessments in new research approvals and fostering research and innovation through dedicated grant funding	25<br>2.6	29<br>2.1	667
	Audit and reduce clinical waste production to <15% of total waste, institute education and behaviour change interventions to reduce clinical waste usage	26<br>2.4	25<br>2.3	650
	Initiate policy to request companies to provide environmental (life-cycle) assessment before purchasing decisions are made	26<br>2.2	25<br>2.5	650
	Implement reusable PPE across all sites	26<br>2.3	25<br>2.5	650
**19**	Source hospital and embedded retail foods from local and sustainable food sources	26<br>2.0	25<br>2.2	650
**19**<br>**20**	Utilise electric vehicles for inter-facility shuttle transport	24<br>2.6	27<br>2.5	648
	Commit to no piped nitrous oxide in future building or capital works upgrades	25<br>2.5	25<br>2.3	625
**21**	Support Choosing Wisely to identify and eliminate low-value clinical care	23<br>2.6	27<br>2.0	621
	Linen services: ensure adequate supply to avoid single-use plastics, assess environmental impact of services and reduce inefficiencies	23<br>2.5	27<br>2.3	621
**22**	Replace fleet vehicles with electric vehicles	28<br>2.3	22<br>2.5	616
**23**	Initiate organic composting waste streams to manage hospital and embedded retail food waste	25<br>2.0	24<br>2.2	600
	Pathology: engage with choosing wisely to reduce unnecessary testing, improve efficiencies, power-down during low-activity	25<br>2.4	24<br>2.4	600
	Promote responsible prescribing: low-carbon prescribing education and audit impacts	25<br>2.2	24<br>2.3	600
**24**	Audit pharmaceutical waste management systems and implement improvements to reduce environmental contamination	23<br>2.3	26<br>2.3	598
**25**	Provide EV charging sites in car parking areas	22<br>2.4	27<br>2.1	594
	Expand single-stream recycling activities in critical care areas such as metal recycling, soft plastics recycling, PVC recycling, kimguard (surgical wrap)	22<br>2.5	27<br>1.9	594

**26**	Remove environmentally harmful pharmaceuticals from formulary, where possible	23<br>2.1	25<br>2.5	575
**27**	Install solar photovoltaic panels on Austin health buildings	27<br>2.1	21<br>2.5	567
**28**	Publicise opportunities for waste to be repurposed when it will otherwise be discarded. For example collecting cardboard boxes for personal use (moving house), reusing batteries used once in disposable theatre equipment	21<br>2.4	26<br>1.8	546
**29**	Work with Indigenous partners to ensure practices are safe and inclusive	20<br>2.2	27<br>1.7	540
**30**	Aim for minimum 5% of renewable energy purchasing until 2025 (when renewable electricity will be supplied by Victorian Government)	26<br>2.0	20<br>2.5	520
**31**	Minimise waste transport: Assess waste providers and consider provider that has best environmental practices	23<br>1.8	22<br>2.1	506
**32**	Develop initiatives that promote oral medications in place of IV, where clinically suitable	21<br>2.2	24<br>2.0	504
**33**	Incentivise and promote cycling to work by upgrading facilities	20<br>2.4	25<br>1.9	500
**34**	Install hot water heat pumps (electric) to replace gas boilers	27<br>1.9	18<br>2.5	486
	Upgrade building insulation to improve building energy efficiency	27<br>1.8	18<br>2.6	486
**35**	Work with community liaison / consumer engagement to explore partnering with consumers in low carbon care	22<br>1.7	22<br>2.0	484
**36**	Reduce water waste with low-flow taps, low-flush toilets etc.	20<br>2.3	24<br>2.0	480
**37**	Prioritise the development of green spaces within the organisation, highlighting the link between health and access to nature as well as promoting staff wellbeing, recruitment and retention	19<br>2.0	25<br>1.5	475
**38**	Explore opportunities to use patient transfer providers with electric vehicles/hybrid vehicles	21<br>2.0	22<br>1.8	462
	Invest in sustainable and low-carbon financial investments	22<br>1.8	21<br>1.8	462
**39**	Audit energy purchases for privately leased buildings and source from renewable energy	21<br>2.0	20<br>1.9	420
**40**	Include rainwater capture and use in new capital works, and explore ability to retrofit to existing infrastructure	19<br>2.1	22<br>1.9	418
**41**	Allow E-bike purchases to be salary packaged	16<br>2.2	26<br>1.5	416
**42**	Introduce strategies to reduce business air travel	16<br>1.0	23<br>1.5	368
**43**	Start or expand grey water recycling (i.e. laundry water and dialysis water recycling)	17<br>1.5	17<br>1.9	289
**44**	Promote public transport by working with local governments to improve timings of services, routes and access to stops/stations	19<br>1.5	15<br>1.8	285
